# Silence of MCL-1 upstream signaling by shRNA abrogates multiple myeloma growth

**DOI:** 10.1186/2162-3619-3-27

**Published:** 2014-11-19

**Authors:** Mengchang Wang, Di Wu, Pengbo Liu, Jiusheng Deng

**Affiliations:** Department of Hematology, First Affiliated Hospital, Xi’an Jiaotong University, Xi’an, 710061 China; Department of Global Health, Rollins School of Public Health, Emory University, Atlanta, 30322 USA; Department of Hematology & Medical Oncology, Winship Cancer Institute, School of Medicine, Emory University, Atlanta, 30322 USA

**Keywords:** Multiple myeloma, shRNA, MCL-1, STAT3, PI3K, MAPK

## Abstract

**Objectives:**

Multiple myeloma (MM) is an incurable B-cell cancer with accumulated clonal abnormal plasma cells in bone marrow of patients. MCL-1 (myeloid cell leukemia sequence 1) protein is an anti-apoptotic molecule in MM cells and regulated by pro-inflammatory cytokine IL-6 and downstream signaling molecules STAT3, PI3K and MAPK. The purpose of this study is to investigate the effect of STAT3, PI3K and MAPK gene silence on MCL-1 expression in human MM cells and the consequence of cell survival.

**Methods:**

Lentivirus small hairpin RNA (shRNA) interference techniques were utilized to knock down STAT3, PI3K or MAPK genes. Gene and protein expression was quantified by quantitative real-time PCR and Western Blot. MM cell apoptosis was examined by annexin-V FITC/propidium iodide staining.

**Results:**

Efficient silence of STAT3, PI3K, MAPK1 or MAPK2 gene robustly abrogated IL-6 enhanced MCL-1 expression and suppressed MM cell growth. Silencing STAT3 gene inhibited PI3K expression, silencing PI3K markedly abrogated STAT3 and MAPK production. Inhibition of MAPK2 gene by shMAPK2 suppressed STAT3, PI3K and MAPK1 expression in the cells. Silencing of STAT3, PI3K and MAPK2 together completely blocked MCL-1 expression in MM cells.

**Conclusion:**

There is a syngeneic effect among the three independent STAT3, PI3K and MAPK2 survival-signaling pathways related to MCL-1 expression in MM cells. shRNAs silencing of STAT3, PI3K and MAPK2 together could provide an effective strategy to treat MM.

## Background

Multiple myeloma (MM) is a B-cell blood cancer that characterized with excessive clonal abnormal plasma cells in the bone marrow [1]. It is the most common hematological malignancy in USA. As a B-cell lymphoma-2 (BCL-2) family member, myeloid cell leukemia-1 (MCL-1) plays critical roles in promoting the survival of MM cells [2] as well as a wide range of other cancer cells due to its anti-apoptosis activities [3]. The mechanism by which Mcl-1 blocks the progression of apoptosis is through binding and sequestering the pro-apoptotic BH3-only proteins Bim, PUMA, Noxa, Bak, and Bax [4], preventing pore formation on mitochondrial membrane of MM cells and the release of cytochrome c into the cytoplasm. In clinical and preclinical studies, pan BCL-2 protein inhibitors have been used to treat patients with multiple myeloma [5]. Those inhibitors are small synthetic BH3 mimetic molecules with the potent capability to bind to anti-apoptotic factors. Monoclonal antibodies targeting the malfunctioned plasma cells have been widely used to treat MM patients either alone or combined with chemotherapy [6].

MCL-1 is a short half-life protein. It is overexpressed in cancers cells, which contributes to drug resistance to conventional chemotherapy [7, 8]. The expression of MCL-1 in MM cells is tightly regulated by survival signaling triggered by cytokines and growth factors in bone marrow microenvironment [2, 9, 10]. IL-6 is a key pro-inflammatory cytokine that activates MCL-1 survival signaling upstream molecules including signal transducer and activator of transcription-3 (STAT3), phosphatidylinositol-3 (PI3K), and Mitogen-activated protein kinase (MAPK) [11]. Those molecules modulate three independent signaling pathways that control MCL-1 transcription [2]. Thus, targeting IL-6 triggered MCL-1 modulating molecules offers potential therapeutic approach to treat multiple myeloma. In this study, we investigated the effects of STAT3, PI3K and MAPK RNA interference on IL-6 induced MCL-1 expression in human MM cells and the apoptotic death of the cells.

## Materials and methods

### Cell culture

Human myeloma cell lines 8226 and U266 were cultured in RPMI-1640 medium (Hyclone, USA) supplemented with 10% heat-inactivated fetal bovine serum (FBS) (Gibco, USA) and penicillin/streptomycin antibiotics at 37°C in a 5% CO_2_ incubator. 293 T cells were cultured in DMEM medium with 10% FBS and penicillin/streptomycin. For cell proliferation assay, U266 or 8226 cells (5,000 cells/well) were cultured in a 96-well plate with 100ul of RPMI-1640 medium for 24 or 48 hours in presence of IL-6 (5 ng/ml) (R&D, USA).

### Gene silencing

STAT3, PI3K and MAPK targeting oligonucleotide sequences were chemically synthesized, used for the cloning of shRNA-encoding sequences, and inserted into a lentiviral expression vector pGV217 (GeneChem, Shanghai, China). STAT3: 5′-GGAGCAGCACCTTCAGGATAA-3′, PI3K: 5′-CAAGATAT GCTAACAC TTCAA-3′, MAPK1: 5′-GACCGGATAACCTTTAAA-3′, MAPK2: 5′-CACCACCTGTGATCTCAAGAA-3′, Control RNA: 5′-UUCUCCGAACGUGUC ACGUTT-3′. The lentiviral vector-bearing shRNAs were confirmed by DNA sequencing. Forty-eight hours after co-transfection with pHelper 1.0 and pHelper 2.0 lentiviral packing plasmid (GeneChem) into 293 T packaging cells using Lipofectamine 2000 (Invitrogen, USA), the lentivirus-containing supernatant was harvested and used for infection of human U266 and 8226 multiple myeloma cells in presence of 4 μg/mL polybrene (Sigma-Aldrich, St. Louis, MO, USA).

**Table 1:** **Primer sequences for qRT-PCR**

Genes	Forward	Reverse
STAT3	5′-ctacagtgacagcttcccaatg-3′	5′-ttggcttctcaagatacctgct-3′
PI3K	5′-acttattgaggtggtgcgaaat-3′	5′-tgatgtagtgtgtggctgttga-3′
MAPK1	5′-ggagaactgaaggatgacgact-3′	5′-gctgtagaacgcaccatagaag-3′
MAPK2	5′-ctcaccatcaaccctaccatc-3′	5′-cttctgctgctcgtcaagttc-3′
MCL-1	5′-ttaaacaaagaggctgggatg-3′	5′-accagctcctactccagcaa-3′
18S rRNA	5′-cagccacccgagattgagca-3′	5′-tagtagcgacgggcggtgtg-3′

### Quantitative Real-time polymerase chain reaction (qRT-PCR)

Total RNA was extracted from cells as previously described [12]. PrimeScript® RT reagent Kit and SYBR® Premix^Ex^ Taq™ II kit were obtained from TAKARA (Dalian, China) and used for conversion of total RNA to cDNA and for the detection of mRNA expression. Each PCR reaction mixture (20 μl) included 10 μl of SYBR Green Master Mix, 0.4 μl of sense and anti-sense primers, and 10 ng of cDNA. The PCR reaction mix was first denatured at 95°C for 10 minutes. PCR was then run for 40 cycles: denaturation at 95°C for 15 seconds and annealing at 60°C for 1 minute. The primer sequences for qRT-PCR were purchased from AuGCT Corporation as listed in Table 1. According to the manufacturer’s instructions, the fold change with 2^-ΔΔCt^ method was used in qRT-PCR data analysis and β-actin was served as a control. All reactions were run in triplicate using the IQ-5 Real-Time PCR System (Bio-Rad, USA).

### Cell apoptotic assay

U266 or 8266 cells (10^6^ cells/well) were cultured in 12-well plates in triplicate and transfected with targeted shRNA against STAT3, PI3K, MAPK1 or MAPK2, or control shRNAs vectors for 48 h following the manufacturer’s instructions. Apoptotic and viable cells fractions were assessed with annexin-V FITC/propidium iodide staining (Invitrogen, USA) on a flow cytometer (Becton, USA) following the manufacturer’s instruction. The apoptotic populations were determined by ModFit software. Alternatively, The cells were harvested and washed in PBS, followed by fixation with ice-cold ethanol overnight. The cells were then washed in PBS and incubated in 1 ml staining solution (20 ug/ml propidium iodide and 10 U/ml RNaseA) for 30 min at room temperature. The cell cycle distributions were assayed by fluorescence-activated cell sorting using a flow cytometer.

### MTT assay

Cell growth was assessed with the 3-(4, 5-dimethyl-2-thiazolyl)-2,5-diphenyl-2H-tetrazolium bromide (MTT) assay. The cells were seeded into wells of a 96-well plate and infected with lentivirus carrying shRNA for 24 and 48 hours at 37°C. The cells were then treated with MTT solution (15 μl/well) for 4 hours at 37°C. After gentle removal of culture supernatant, the cell pellets were dissolved with 200 μl of Dimethyl sulfoxide (DMSO). Optical density (OD) at 570 nm was read on a FLUOstar OPTIMA machine (BMG Labtech). Experiments were performed in triplicate.

### Western blot

U266 or 8266 cells were harvested 24 or 48 hours after IL-6 treatment and washed with cold PBS. Total proteins extracted from the cells using a RIPA cell lysis buffer (Wolsen, China) were separated on 10% SDS polyacrylamide gels and electrophoretically transferred to polyvinylidene difluoride membrane (Millipore, USA). The membranes were treated with mouse anti-STAT3, rabbit anti-MCL-1, anti-PI3K or anti-MAPK1/2 antibodies, rabbit anti-pSTAT-3, anti-pMAPK1/2 antibodies, and mouse anti-β-actin antibodies (1:1000 dilution) (Cell Signaling Technology, USA). The membranes were then reacted with the HRP-conjugated secondary antibodies before subjected to enhanced chemiluminescent (ECL) detection on an ECL machine (Pierce, USA). The blots were scanned and the band density was measured on the Quantity One imaging software.

### Statistical analysis

All data were presented as mean ± SD (standard deviation). The MCL-1 protein expression levels post IL-6 treatment, cell growth, and percentage of apoptotic cells in different shRNA silencing were analyzed by One-way ANOVA. *P* < 0.05 was considered statistically significant (*, *P* < 0.05; **, *P* < 0.01).

## Results

### IL-6 stimulation enhanced MCL-1 expression and myeloma growth

**Figure 1:**
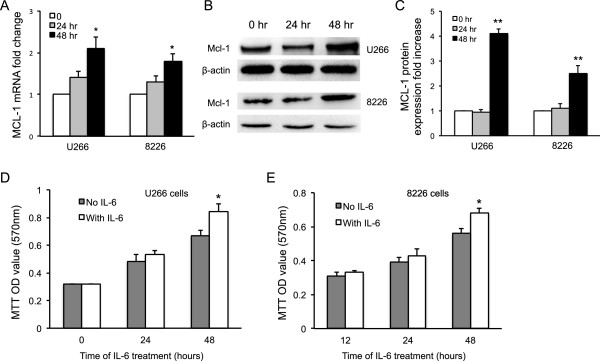
**IL-6 enhanced MCL-1 expression and MM cell growth.** Human U266 and 8226 MM cells were cultured in RPMI and stimulated with IL-6 for 0, 24 or 48 hours. **(A)** MCL-1 gene expression in the cells was quantified by qRT-PCR. mRNA fold change was presented. **(B)** MCL-1 protein expression was detected by Western blot with anti-MCL-1 specific antibodies. **(C)** MCL-1 protein expression in IL-6 treated group was quantified based on β-actin level and in comparison with untreated cells. **(D** **and E)** U266 and 8226 MM cell growth after IL-6 treatment was measured by MTT assay. Cell growth was presented as OD values. Data represents three independent experiments. *, *P* < 0.05; **, *P* < 0.01.

To evaluate the effect of pro-inflammatory cytokine IL-6 on MCL-1 expression and myeloma growth, human U266 and 8226 multiple myeloma cells were treated with or without recombinant cytokine IL-6 *in vitro*. Total RNA and protein were isolated from the treated cells. qRT-PCR assay revealed that IL-6 treatment significantly enhanced MCL-1 gene expression in both U266 and 8226 cells after 48-hour culture compared to the control without treatment (*p* < 0.05) (Figure 1A); there was a slight increase of MCL-1 in the 24-hour treatment of IL-6, but not significant. Western blot with anti-human MCL-1 monoclonal antibodies showed that IL-6 treatment (48 hours) significantly enhanced MCL-1 protein expression levels (Figure 1B), with a 4 fold increase in U266 cells and a 2.5 fold increase in 8226 cells in comparison with no IL-6 treatment control or 24 hours treatment (*p* < 0.01) (Figure 1C). While, MCL-1 levels showed no difference between 24-hour and no IL-6 treatment. Consistently, IL-6 treatment significantly enhanced the growth of myeloma cells after 48-hour treatment with IL-6 in U266 cell line (Figure 1D) and 8226-cell line, in comparison with the treatment without IL-6 (*p* < 0.05) (Figure 1E).

### Silencing STAT3, PI3K, MAPK1 or MAPK2 abrogated IL-6 triggered MCL-1 expression in human MM cells

**Figure 2:**
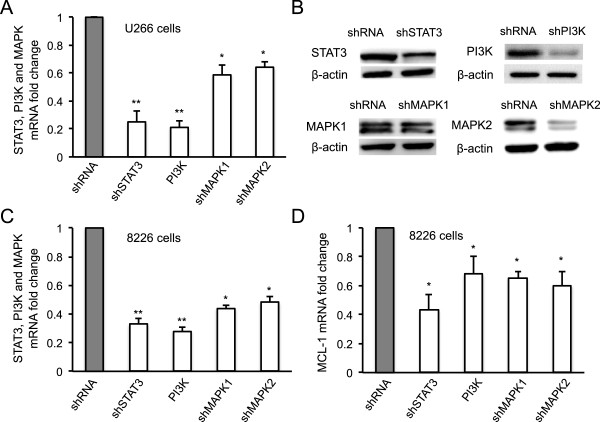
**Effect of shSTAT3, shPI3K and shMAPK1/2 on MCL-1 gene expression.** Total RNA was isolated from human U266 and 8226 cells transfected with lentivirus carrying shSTAT3, PI3K shMAPK1, shMAPK2 or control shRNA in presence of IL-6 (48 hours). **(A)** mRNA expression of STAT3, PI3K and MAPK in U266 cells were quantified by qRT-PCR. The mRNA levels were presented as fold change in comparison with the control shRNA treatment group. **(B)** Protein expression of STAT3, PI3K or MAPK in U266 cells was determined by Western blot with specific antibodies against the protein. **(C)** mRNA levels of STAT3, PI3K and MAPK in 8226 cells were also quantified by qRT-PCR and presented as fold changes compared with the shRNA treatment control group. **(D)** MCL-1 mRNA expression in 8226 cells was quantified by qRT-PCR. The fold change of MCL-1 mRNA in targeted cells was compared with the control shRNA treatment group. *, *P* < 0.05; **, *P* < 0.01.

To examine whether silencing MCL-1 upstream signaling modulators STAT3, PI3K and MAPK could abrogate IL-6 triggered MCL-1 expression in myeloma cells, we constructed lentiviral GV217 vectors carrying small hairpin RNAs (shRNA) [13] for STAT3 (shSTAT3), PI3K (shPI3K) and MAPK1/2 (shMAPK1 or shMAPK2), and transfected the lentiviral vectors into myeloma cells. Quantification of mRNA levels by qRT-PCR demonstrated that shSTAT3 and shPI3K markedly reduced STAT3 and PI3K mRNA expression in U266 myeloma cells compared to control shRNA treatment (*p* < 0.01); shMAPK1 and shMAPK2 significantly reduced MAPK expression in the cells in comparison with control shRNA treatment (*p* < 0.05) (Figure 2A). Western blot confirmed that silencing mRNA expression markedly reduced STAT3, PI3K and MAPK protein expression levels in U266 cells (Figure 2B). The abrogative effects of small hairpin silencing RNA on targeted STAT3, PI3K and MAPK gene expressions were also observed in 8226 myeloma cells in comparison with control shRNA treatment (Figure 2C). Accordingly, MCL-1 gene expression was significantly reduced by shSTAT3, shPI3K, shMAPK1 or shMAPK2 (*p* < 0.05) (Figure 2D).

### Interactions among STAT3, PI3K and MAPK signaling on IL-6 induced MCL-1 gene expression in myeloma cells

**Figure 3:**
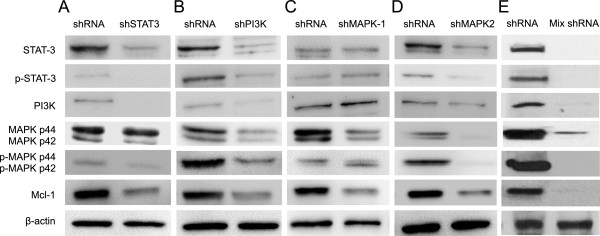
**Interaction among STAT3, PI3K and MAPK.** Total protein was isolated from IL-6 treated human 8226 cells transfected with lentivirus bearing shSTAT3 **(A)**, shPI3K **(B)**, shMAPK1 **(C)**, shMAPK2 **(D)**, or combined shSTAT3 + shPI3K + shMAPK2 **(E)**, and shRNA control. The proteins were profiled by Western blot with specific antibodies against STAT3, pSTAT3, PI3K, MAPK, pMAPK, MCL-1 or β-actin. Data represented one of two independent experiments.

To explore the potential interactions among the three signaling molecules on MCL-1 expression in IL-6 treated myeloma cells, the protein expressions of STAT3, PI3K, MAPK and MCL-1 was profiled by Western blot in human 8226 myeloma cells transfected with shSTAT3-, shPI3K-, shMAPK1- or shMAPK3-bearing lentivirus. In comparison with control shRNA, silencing STAT3 protein expression by shSTAT3 completely blocked PI3K protein expression and markedly reduced p-ERK1/2, consequently suppressed the expression of the downstream MCL-1 protein in the IL-6 treated 8226 cells. However, no inhibitory effect was observed on total MAPK protein expression (Figure 3A). We also observed that silencing PI3K with shPI3K significantly reduced the protein levels of total STAT3, pSTAT3, total MAPK, the phosphorylation of MAPK p44, and downstream MCL-1 (Figure 3B). Compared with control shRNA treatment, silencing MAPK1 with shMAPK1 robustly suppressed MCL-1 expression, but did not affect the protein level of STAT3 and PI3K (Figure 3C). When silencing MAPK2 expression in 8226 cells, there was a remarkable decrease of STAT3 protein expression in treated cells in comparison with shRNA control group (Figure 3D). There was also a slight reduction of PI3K expression in shMAPK2 treated 8226 cells. Moreover, silencing STAT3, PI3K and MAPK2 at the same time completely blocked STAT3, PI3K, MAPK2 expression, with little MAPK1 expression, leading to the absence of MCL-1 production in MM cells (Figure 3E). Taken together, there was an interaction among STAT3, PI3K and MAPK2 signaling pathways.

### STAT3, PI3K and MAPK RNA interference abrogated myeloma cell growth

**Figure 4:**
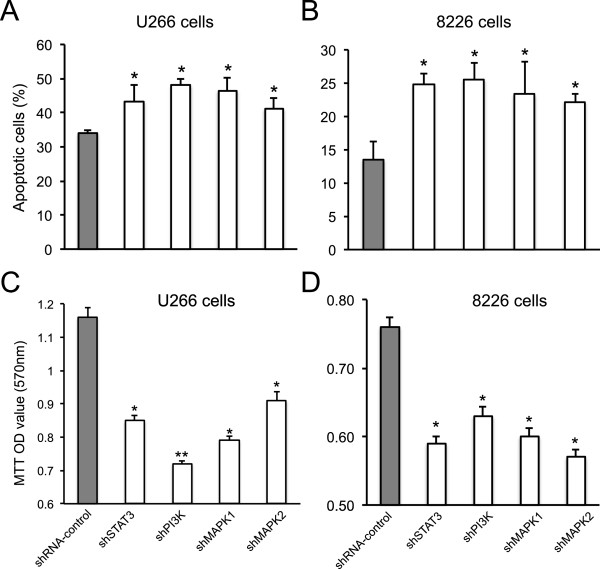
**Effect of STAT3, PI3K and MAPK gene silencing on MM cell apoptosis and cell growth**. Lentirirus-transfected human U266 and 8226 MM cells (bearing shSTAT3, shPI3K, shMAPK or control shRNA) were cultured in RPMI-1640 medium in presence of IL-6 for 48 hours. **(A and** **B)** The cells were harvested and subjected to apoptotic analyses. The percentage of apoptotic cells in U266 or 8226 cells was presented. **(C and** **D)** The cell growth was measured by MTT assay and presented as absorbance values at 570 nm. Data were represented from three independent experiments. *, *P* < 0.05; **, *P* < 0.01.

To analyze the effect of STAT3, PI3K and MAPK gene silencing on the apoptosis of targeted myeloma cells, IL-6 stimulated human U266 or 8226 cells were harvested 48 hours after shRNA-bearing lentivirus treatment. The cells were stained with annexin-V and propidium iodide. Flow cytometry analysis showed that silencing STAT3, PI3K, or MAPK1 or MAPK2 significantly promoted cell apoptosis in U266 cells as well as in 8226 cells, compared to control shRNA treatment (*p* < 0.05) (Figure 4A and 4B). In U266 cells, targeted shRNA treatment induced an average of 44-49% cell apoptosis. While control shRNA group only showed a 33% cell apoptosis. In 8226 cells, shSTAT3, shPI3K and shMAPK treatments led to a 23-26% cell apoptosis compared with only a 13% cell apoptosis in the shRNA control group (*p* < 0.05). Consequently, targeted shRNA treatments markedly inhibited the growth of myeloma cells in both cell lines in comparison with control shRNA treatments (Figure 4C and D).

## Discussion

The findings in this study indicated that RNA silence of STAT3, PI3K, or MAPK gene using shRNA interference robustly inhibited MCL-1 expression in IL-6 treated human MM cells, leading to remarkable myeloma cell apoptosis. MCL-1 plays critical roles in cancer cell survival and drug resistance against chemotherapy [8, 14, 15] and become a promising therapeutic target for MM treatment [16]. RNA silencing/interference is a technique that can specifically and efficiently knocks down target genes. STAT3, PI3K and MAPK are three key upstream signaling molecules that control MCL-1 expression. It has showed selective targeting MCL-1 or its upstream modulatory molecules by RNA interference induced cancer cell death [17–20]. Our study extends the previous studies using shRNA gene interference technique for targeting oncogenes in MM cells. To our knowledge, it is the first report showing that efficient knockdown of PI3K or MAPK gene by shRNA could sufficiently block MCL-1 expression in MM cells and resulted in consequent cell death, which offers a potential to utilize shRN/RNA interference as an alternative of chemotherapy for treat drug-resistant MM treatment.

RNA interference technique has been widely used to investigate the functional interaction among related genes by selective silence of one gene. In this study, we also assessed the mutual effect of shRNA silencing among STAT3, PI3K and MAPK genes. It was reported that STAT3 had functional activities in PI3K-driven oncogenic transformation and there was a crosstalk between STAT3 and PI3K signaling pathways in driving the transformation of murine glioblastoma [21] and lymphoblastic B-cell lymphomas [22], and caused drug resistance [23]. Qiang et al. reported that there is a crosstalk between PI3K and MAKP pathways in MM cells [24]. Consistent with their study, we found that silencing MAPK2 but not MAPK1 had marked suppressive effect on PI3K as well as STAT3. MAPK1/2 signaling network involves many cross-talk nodes and routes interactive with other cell survival signaling pathways such as PI3K [25], it is not surprising that silencing MAPK1 and MAPK2 could have distinct effects on PI3K and STAT3 expression in MM cells. Collectively, our data demonstrated that knockdown one of STAT3, PI3K and MAPK2 genes likely affected the expression of the other two genes in human MM cells and support the evidence that blockade of both PI3K and MAPK pathways caused a significantly higher percentage of human primary MM cell death than individual inhibition [26]. These data suggest that combined targeting IL-6-activated STAT3, PI3K and MAPK molecules or signaling pathways tend to provide better clinical treatment for MM patients, considering the heterogeneous phenotypes of MM and the emerging drug resistance in the course of chemotherapy [27].

In conclusion, we have efficiently delivered lentivirus shRNA to knock down STAT3, PI3K, MAPK genes and downstream MCL-1 expression in human MM cells, and increased cell apoptosis. Selectively silence of MCL-1 upstream signaling molecules STAT3, PI3K and MAPK by shRNA provides a potent strategy to treat drug-resistance human MM.
